# Supplementary material for: Superconductivity in kagome metals due to soft loop-current fluctuations
Source: Nat Commun. 2026 May 21;17:4557. doi: 10.1038/s41467-026-72806-w (PMC13194700; doi:10.1038/s41467-026-72806-w)
Supplement: Supplementary file 1 — Supplementary Information [file 41467_2026_72806_MOESM1_ESM.pdf]

Supplementary to "Superconductivity in kagome metals due to soft loop-current fluctuations"

Daniel J. Schultz,[1] Grgur Palle,[2,3] Asimpunya Mitra,[4] Yong Baek Kim,[4] Rafael M. Fernandes,[2,3] and Jörg Schmalian[1,5]

[1]*Institute for Theoretical Condensed Matter Physics, Karlsruhe Institute of Technology, 76131 Karlsruhe, Germany*
[2]*Department of Physics, The Grainger College of Engineering, University of Illinois Urbana-Champaign, Urbana, Illinois 61801, USA*
[3]*Anthony J. Leggett Institute for Condensed Matter Theory, The Grainger College of Engineering, University of Illinois Urbana-Champaign, Urbana, Illinois 61801, USA*
[4]*Department of Physics, University of Toronto, Toronto, Ontario M5S 1A7, Canada*
[5]*Institute for Quantum Materials and Technologies, Karlsruhe Institute of Technology, 76131 Karlsruhe, Germany*

(Dated: April 3, 2026)

S1. (REDUCED) SPACE GROUP TRANSFORMATIONS

AV_3Sb_5 has the space group P6/mmm. This is a symmorphic group, and thus may be written as a semidirect product of the D_{6h} point group with the group of lattice translations. Since we are focusing on a single layer, we ignore translations in the z (out of plane) direction. We define the x and y (and other equivalent) rotation axes in Fig. S1. The z-axis points out of the page.

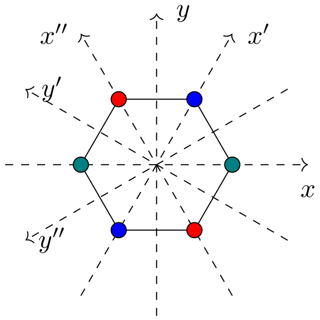

FIG. S1. In-plane rotation axes for the C_2 rotations. The hexagon orientation pictured here is the same hexagon appearing in the kagome lattice.

The group theory classification in this work concerns loop current patterns living in the 2×2 extended unit cell, as pictured in Fig. S2. We therefore consider the irreducible representations of a *reduced space group* (sometimes also called extended point group) consisting of four unit cells. These four unit cells contain 12 kagome lattice sites (because there are 3 kagome/vanadium sites per unit cell), and 24 nearest neighbour bonds. In addition to the 24 point group operations of D_{6h}, we may now perform translations by $\boldsymbol{a}_1 = (1, 0)$, $\boldsymbol{a}_2 = (\frac{1}{2}, \frac{\sqrt{3}}{2})$, and $\boldsymbol{a}_1 + \boldsymbol{a}_2$. Within this reduced space group, a translation by $2\boldsymbol{a}_1$ or $2\boldsymbol{a}_2$ is the same as the identity operation. The result is a group of 96 elements. The character table and conjugacy classes for this group are outlined in Table I. We remark that σ_h is the operation which sends $z \to -z$, and the other mirror reflections are $\sigma_v : y \to -y$ and $\sigma_d : x \to -x$. These other mirror reflections may alternatively be written as $\sigma_v = I \circ C_{2y}$ and $\sigma_d = I \circ C_{2x}$.

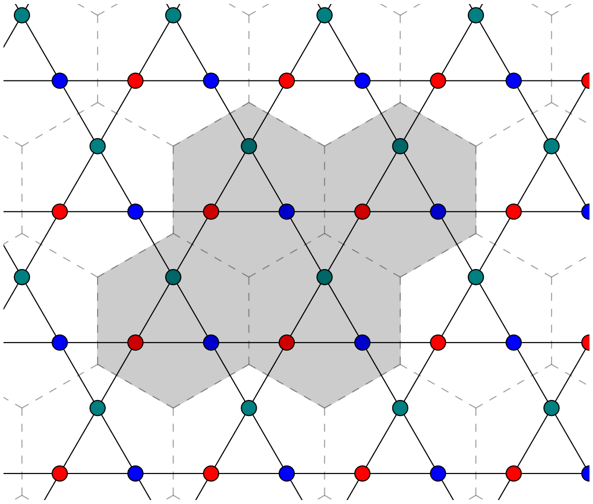

FIG. S2. Extended unit cell. All loop current patterns live within the greyed out area, and are repeated periodically around the lattice using the translation vectors $2\mathbf{a}_1$ and $2\mathbf{a}_2$. Note that the Sb atoms are not depicted in this figure. Only the V atom lattice sites are shown here.

Irrep	E	C_{6z}	C_{3z}	C_{2z}	C_{2x}	C_{2y}	I	S_6	S_3	σ_h	σ_v	σ_d	T	C_{2z}^t	C_{2x}^t	C_{2y}^t	I^t	σ_h^t	σ_v^t	σ_d^t	Basis functions
A_{1g}	1	1	1	1	1	1	1	1	1	1	1	1	1	1	1	1	1	1	1	1	$\{\lvert z^2\rangle\}$
A_{2g}	1	1	1	1	−1	−1	1	1	1	1	−1	−1	1	1	−1	−1	1	1	−1	−1	$\{\lvert xy(x^2-3y^2)(3x^2-y^2)\rangle\}$
B_{1g}	1	−1	1	−1	1	−1	1	1	−1	−1	−1	1	1	−1	1	−1	1	−1	−1	1	$\{\lvert yz(x^2-3y^2)\rangle\}$
B_{2g}	1	−1	1	−1	−1	1	1	1	−1	−1	1	−1	1	−1	−1	1	1	−1	1	−1	$\{\lvert zx(3x^2-y^2)\rangle\}$
E_{1g}	2	1	−1	−2	0	0	2	−1	1	−2	0	0	2	−2	0	0	2	−2	0	0	$\{\lvert zx\rangle, \lvert yz\rangle\}$
E_{2g}	2	−1	−1	2	0	0	2	−1	−1	2	0	0	2	2	0	0	2	2	0	0	$\{\lvert x^2-y^2\rangle, \lvert xy\rangle\}$
A_{1u}	1	1	1	1	1	1	−1	−1	−1	−1	−1	−1	1	1	1	1	−1	−1	−1	−1	$\{\lvert xyz(x^2-3y^2)(3x^2-y^2)\rangle\}$
A_{2u}	1	1	1	1	−1	−1	−1	−1	−1	−1	1	1	1	1	−1	−1	−1	−1	1	1	$\{\lvert z\rangle\}$
B_{1u}	1	−1	1	−1	1	−1	−1	−1	1	1	1	−1	1	−1	1	−1	−1	1	1	−1	$\{\lvert x(x^2-3y^2)\rangle\}$
B_{2u}	1	−1	1	−1	−1	1	−1	−1	1	1	−1	1	1	−1	−1	1	−1	1	−1	1	$\{\lvert y(3x^2-y^2)\rangle\}$
E_{1u}	2	1	−1	−2	0	0	−2	1	−1	2	0	0	2	−2	0	0	−2	2	0	0	$\{\lvert x\rangle, \lvert y\rangle\}$
E_{2u}	2	−1	−1	2	0	0	−2	1	1	−2	0	0	2	2	0	0	−2	−2	0	0	$\{\lvert z(x^2-y^2)\rangle, \lvert xyz\rangle\}$
M_1^+	3	0	0	3	1	1	3	0	0	3	1	1	−1	−1	−1	−1	−1	−1	−1	−1	
M_2^+	3	0	0	3	−1	−1	3	0	0	3	−1	−1	−1	−1	1	1	−1	−1	1	1	
M_3^+	3	0	0	−3	1	−1	3	0	0	−3	−1	1	−1	1	−1	1	−1	1	1	−1	
M_4^+	3	0	0	−3	−1	1	3	0	0	−3	1	−1	−1	1	1	−1	−1	1	−1	1	
M_1^-	3	0	0	3	1	1	−3	0	0	−3	−1	−1	−1	−1	−1	−1	1	1	1	1	
M_2^-	3	0	0	3	−1	−1	−3	0	0	−3	1	1	−1	−1	1	1	1	1	−1	−1	
M_3^-	3	0	0	−3	1	−1	−3	0	0	3	1	−1	−1	1	−1	1	1	−1	−1	1	
M_4^-	3	0	0	−3	−1	1	−3	0	0	3	−1	1	−1	1	1	−1	1	−1	1	−1	

TABLE I. Character table of the reduced space group, corresponding to D_{6h} plus translations modulo $2\boldsymbol{a}_1$ and $2\boldsymbol{a}_2$. Note that, if we are talking about fermion bilinears (like loop current operators), then we can also classify the operators according to their character under time-reversal, and we insert an m, e.g. mA_{2g} to denote a time-reversal odd irrep, which spatially transforms like A_{2g}.

S2. CURRENT OPERATORS AND THE CURRENT REPRESENTATION

A. Derivation of current operator

The local current operator for a charge-conserving (global U(1) phase-rotation symmetric), time-reversal symmetric, lattice model can be found by considering the number density operator $\hat{n}_i = \sum_{a\sigma} \hat{c}^\dagger_{ia\sigma}\hat{c}_{ia\sigma}$, whereby i is a lattice site, and a labels orbitals on that site. The time-dependent operator $\hat{n}_i(t) = e^{i\hat{H}t}\hat{n}_i e^{-i\hat{H}t}$ satisfies the Heisenberg equation of motion

$$\frac{\mathrm{d}\hat{n}_i(t)}{\mathrm{d}t} = i[\hat{H}, \hat{n}_i(t)]. \tag{S1}$$

We then use the conservation law $\mathrm{d}\hat{n}_i/\mathrm{d}t + (\nabla\cdot\boldsymbol{j})_i = 0$. Note that the discretized divergence is given by

$$(\nabla\cdot\hat{\boldsymbol{j}})_i = -\sum_j \hat{j}_{ij}, \tag{S2}$$

where $\hat{j}_{ij}$ represents the current flowing from site j to site i. Consider a free fermion system described by a hopping Hamiltonian of the form

$$\hat{H} = \sum_{ijab\sigma} \hat{c}^\dagger_{ia\sigma} H_{ijab}\hat{c}_{jb\sigma}, \tag{S3}$$

which must satisfy the relation $H_{ijab} = H^*_{jiba}$ due to Hermiticity. Here, a, b label the different orbitals on the sites i, j respectively. By computing the commutator $[\hat{H}, \hat{n}_{i\sigma}]$, the form of the current operator follows

$$\hat{j}_{ij} = i\sum_{ab\sigma}(-H_{ijab}\hat{c}^\dagger_{ia\sigma}\hat{c}_{jb\sigma} + H_{jiba}\hat{c}^\dagger_{jb\sigma}\hat{c}_{ia\sigma}). \tag{S4}$$

We note that the current operator is odd under swapping the two sites and that it is Hermitian, $\hat{j}_{ij} = -\hat{j}_{ji} = \hat{j}^\dagger_{ij}$. Furthermore, it is odd under the time-reversal operation: $\hat{\mathcal{T}}\hat{j}_{ij}\hat{\mathcal{T}}^{-1} = -\hat{j}_{ij}$. Group theoretical considerations alone are not enough to construct the valid current patterns. Some of the current patterns in the group theory classification are not allowed, due to the following two constraints:

1. Kirchoff constraint: there can be no net current into/out of a particular site. Such a scenario would result in charge buildup on that site. In mathematical terms, $\langle(\nabla\cdot\hat{\boldsymbol{j}})_i\rangle = 0$, for every site i.

2. Bloch constraint: there can be no global current. In mathematical terms, if we define the net current (which is a vector) through a site by $\hat{\boldsymbol{J}}_i = \sum_j \boldsymbol{r}_{ij}\hat{j}_{ij}$ (whereby $\boldsymbol{r}_{ij} = \frac{\boldsymbol{R}_i - \boldsymbol{R}_j}{|\boldsymbol{R}_i - \boldsymbol{R}_j|}$), then the total current must vanish $\sum_i \langle\hat{\boldsymbol{J}}_i\rangle = 0$. This condition is automatically satisfied for any loop current pattern that has a nonzero ordering wave vector.

Some of the loop current patterns generated from the group theoretical analysis violate these constraints, and must therefore be discarded. All patterns that we have presented in the main text obey these two constraints.

B. The current representation

In our model, we have a variety of hoppings into/out of a kagome lattice site, as well as into/out of the in-plane Sb site. There are nearest and next-nearest V hoppings, as well as hybridizations between V and Sb, both in and out of plane. This leads to a huge number of bonds along which hopping can occur, and allowing currents to flow among all of them greatly complicates our analysis. For simplicity, we consider two classes of current patterns:

1. Current patterns that flow only between nearest neighbor V sites. There are 24 such bonds, hence creating a 24 dimensional vector space to represent the reduced space group on. As mentioned in the main text, the tight-binding model can be separated into the $\sigma_h = 1$ and $\sigma_h = -1$ sectors. Since we are considering only $\sigma_h = -1$, we allow only currents between d_{zx}, d_{yz} orbitals. This representation is then reduced as follows:

$$R_{\text{V-V current}} = mA_{2g} \oplus mE_{2g} \oplus mB_{1u} \oplus mE_{1u} \oplus mM_1^+ \oplus mM_2^+ \oplus mM_2^+ \oplus mM_3^- \oplus mM_3^- \oplus mM_4^- \tag{S5}$$

The m prefix indicates oddness under time-reversal. For the first four irreps that have zero momentum (Table I), the g/u subscripts indicate evenness/oddness under spatial inversion, respectively, while for the last six M-point irreps, the $\pm$ superscripts denote the evenness/oddness under spatial inversion. Both copies of mM_2^+ are analyzed in the main text, namely patterns (a) and (b) of Fig. 3. Although there are two copies of mM_3^-, only one fine-tuned linear combination of them satisfies the Kirchhoff constraint and is pictured in pattern (d) of Fig. 3.

2. Current patterns that flow between nearest neighbor V and in-plane Sb sites. There are also 24 such bonds, hence creating a 24 dimensional vector space to represent the reduced space group on. Since we are considering only $\sigma_h = -1$, we only allow currents between d_{zx}, d_{yz} and p_z (from planar Sb) orbitals. The decomposition:

$$R_{\text{V-Sb current}} = mA_{1g} \oplus mE_{2g} \oplus mB_{1u} \oplus mE_{1u} \oplus mM_1^+ \oplus mM_1^+ \oplus mM_2^+ \oplus mM_3^- \oplus mM_3^- \oplus mM_4^- \tag{S6}$$

The one copy of mM_2^+ is shown in the main text, Fig. 3, as pattern (c). The pattern (e) of Fig. 3 is made of mM_3^- that is superimposed (in a way satisfying the Kirchhoff constraint) with a mM_3^- pattern coming from V-V patterns.

C. Current patterns

In this section, we discuss in more detail the current patterns listed in the main text and what is their relationship to other current patterns discussed in the literature. Because all current patterns have one of the M-points as their ordering wave vector, they come in sets of three: one with ordering wave vector $\boldsymbol{M}_1$, one with ordering wave vector $\boldsymbol{M}_2$, and one with ordering wave vector $\boldsymbol{M}_3$; see panel (f) of Fig. 3 of the main text. Only the $\boldsymbol{M}_3$ representative is shown in the main text, but here in Figs. S3 to S7 we also show the other representatives, as well as the corresponding 3-$\boldsymbol{Q}$ orders that are equal-weight $\propto (1,1,1)$ superposition of the three individual 1-$\boldsymbol{Q}$ orders.

For the reader's convenience, below we identify the patterns considered by us with some selected works from the literature.

- The pattern in Fig. 2c of Ref. [1] is in the irrep mM_2^+, and is a weighted superposition of our patterns in Figs. S3 and Figs. S4.
- The patterns in Fig. 2 of Ref. [2] are all in the irrep mM_2^+, and are a weighted superposition of our patterns in Figs. S3 and Figs. S4.
- The four patterns in Fig. 4b–4e of Ref. [3] are all in the irrep mM_2^+. In particular, they are all weighted superpositions of our patterns in Figs. S3 and S4, and their Fig. 4d even appears to be the specific linear combination comprising only our Fig. S4.
- There are four patterns in Fig. 6 of Ref. [4]. In their Fig. 6a (irrep mM_2^+), this is a weighted superposition of our patterns in Figs. S3 and Figs. S4. In their Fig. 6b (irrep mM_2^+), this is our Fig. S3. In their Fig. 6c (irrep mM_2^+), this is a weighted superposition of our patterns in Figs. S3 and Figs. S4. In their Fig. 6d (irrep mM_3^-), this is our Fig. S6.
- The pattern in Fig. 2a of Ref. [5] is in the irrep mM_2^+, and is a weighted superposition of our patterns in Figs. S3 and Figs. S4.

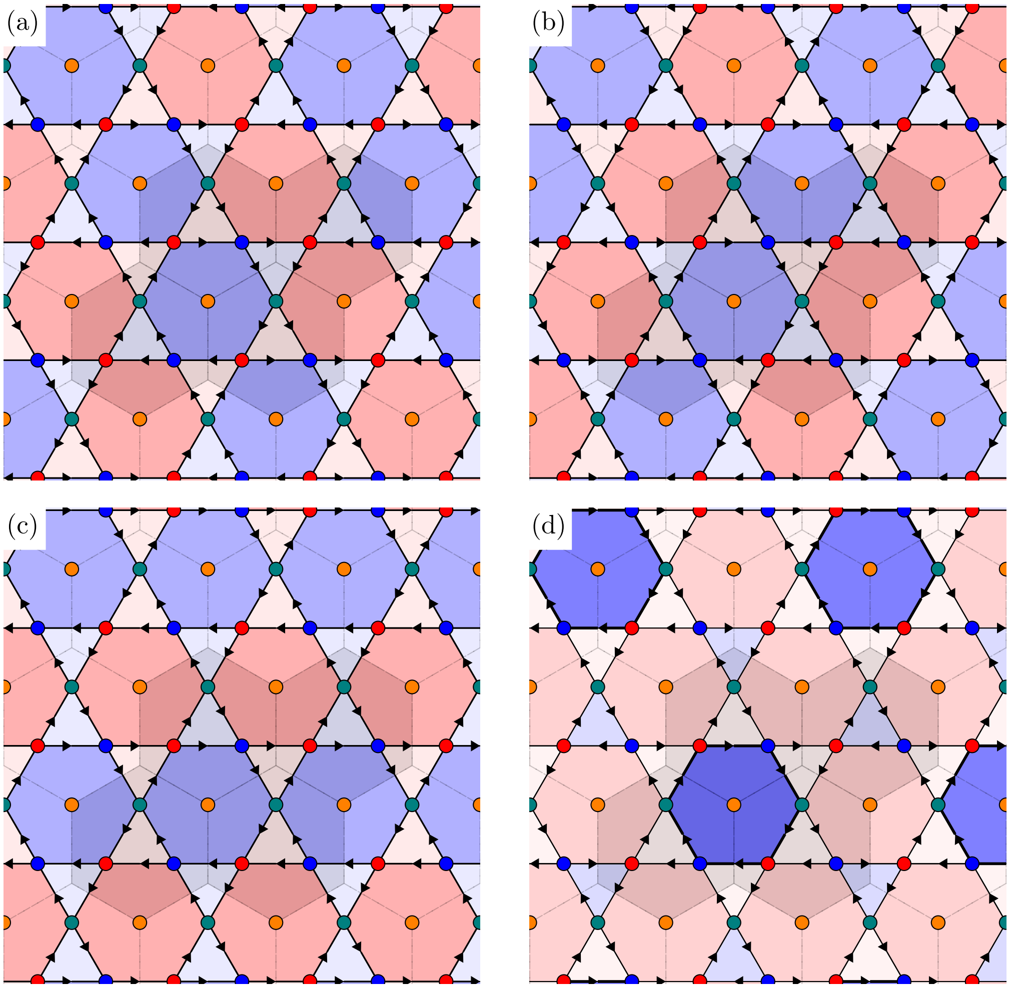

FIG. S3. The current patterns for Fig. 3(a) of the main text. They are in the irrep mM_2^+ and only have currents flowing between V-V. The patterns (a),(b),(c) have ordering wave vectors $\boldsymbol{M}_1$, $\boldsymbol{M}_2$, and $\boldsymbol{M}_3$, respectively. The fourth pattern (d) is the superposition of the three patterns.

.

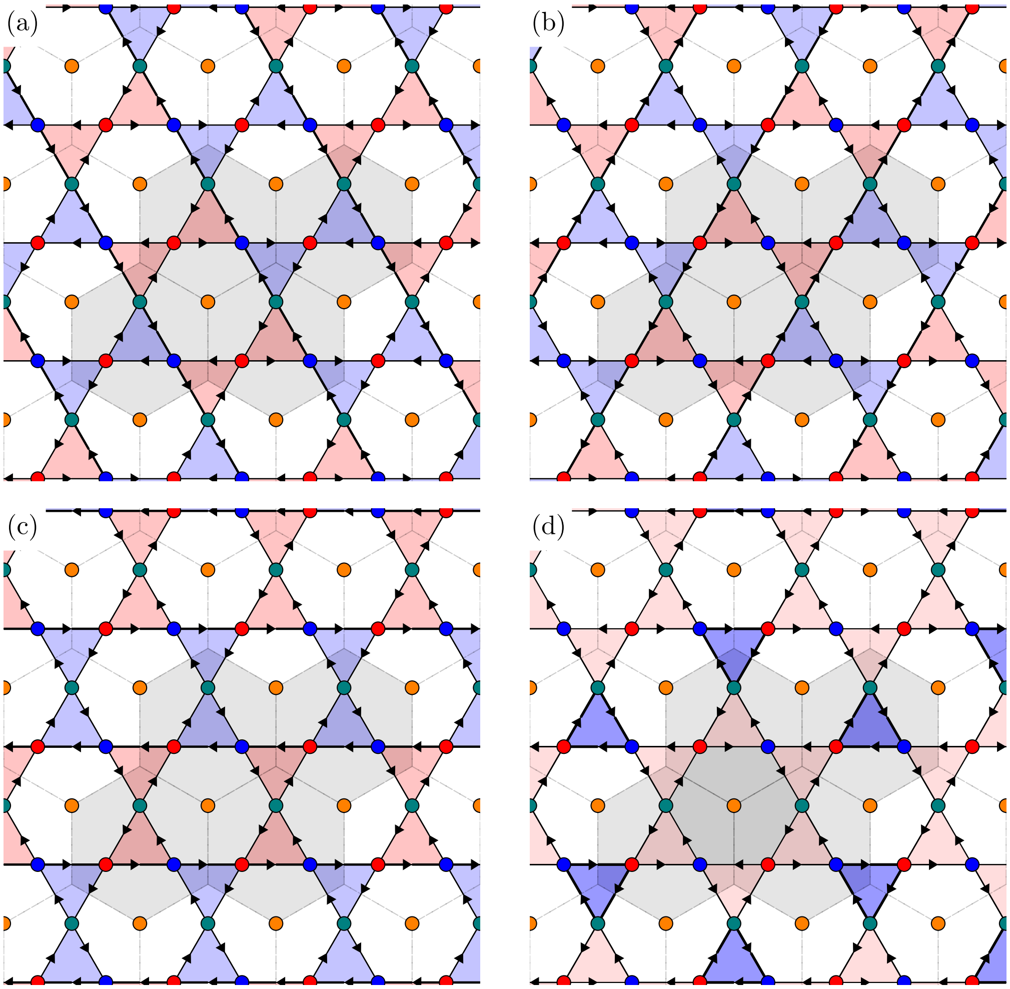

FIG. S4. The current patterns for Fig. 3(b) of the main text. They are in the irrep mM_2^+ and only have currents flowing between V-V. The patterns (a),(b),(c) have ordering wave vectors $\boldsymbol{M}_1$, $\boldsymbol{M}_2$, and $\boldsymbol{M}_3$, respectively. The fourth pattern (d) is the superposition of the three patterns.

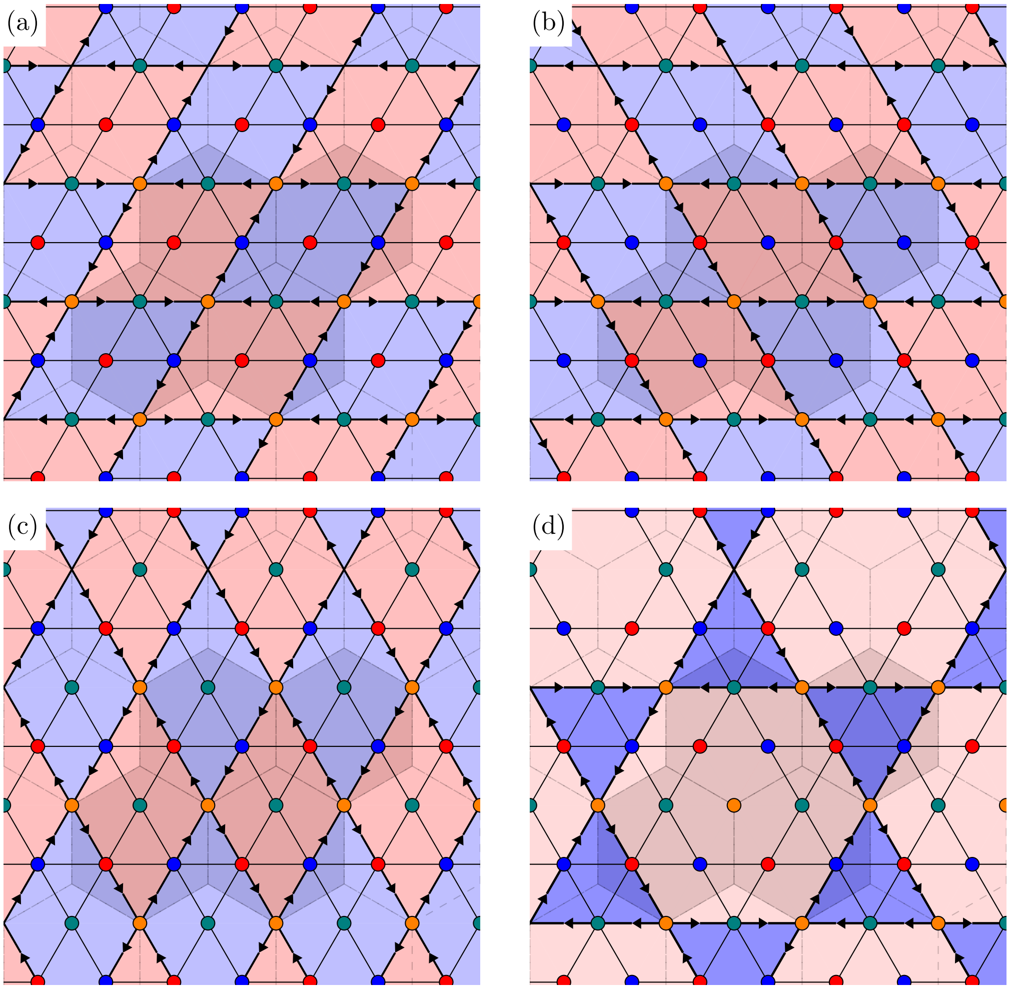

FIG. S5. The current patterns for Fig. 3(c) of the main text. They are in the irrep mM_2^+ and only have currents flowing between V-Sb. The patterns (a),(b),(c) have ordering wave vectors $\boldsymbol{M}_1$, $\boldsymbol{M}_2$, and $\boldsymbol{M}_3$, respectively. The fourth pattern (d) is the superposition of the three patterns.

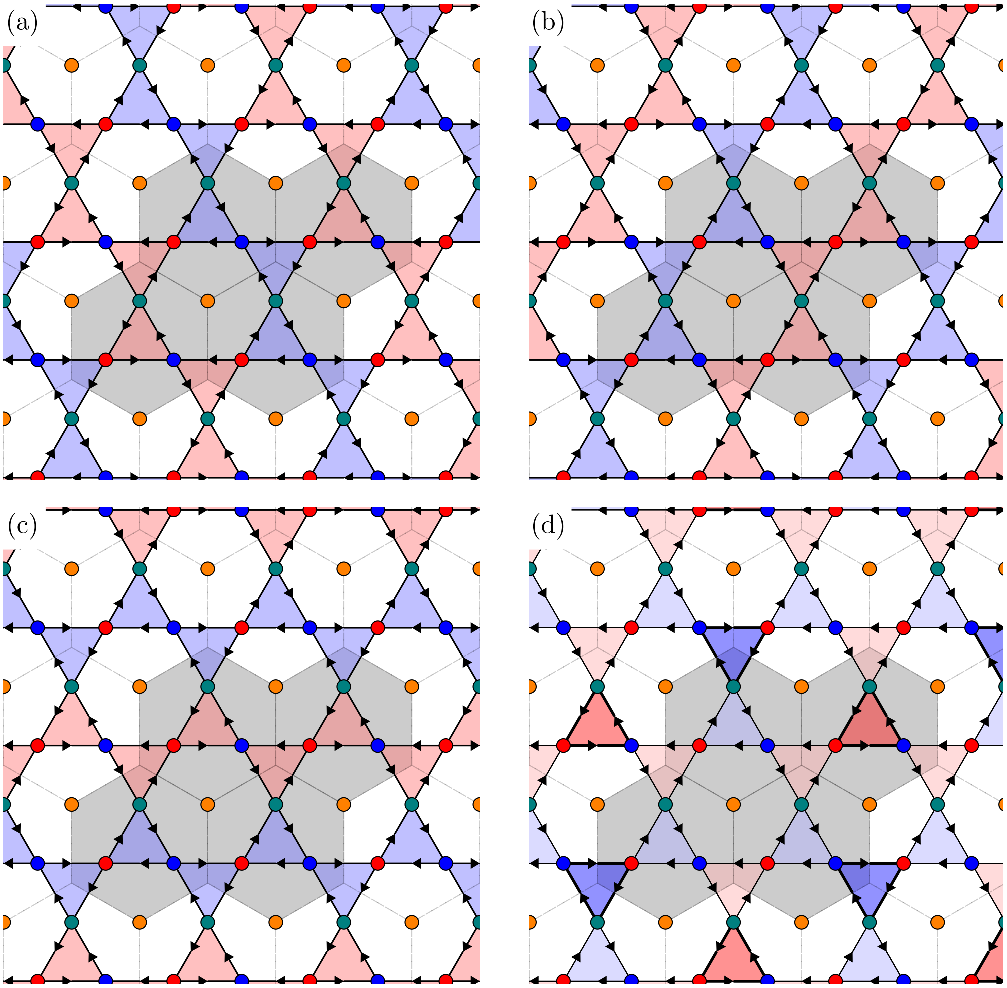

FIG. S6. The current patterns for Fig. 3(d) of the main text. They are in the irrep mM_3^- and only have currents flowing between V-V. The patterns (a),(b),(c) have ordering wave vectors $\boldsymbol{M}_1$, $\boldsymbol{M}_2$, and $\boldsymbol{M}_3$, respectively. The fourth pattern (d) is the superposition of the three patterns.

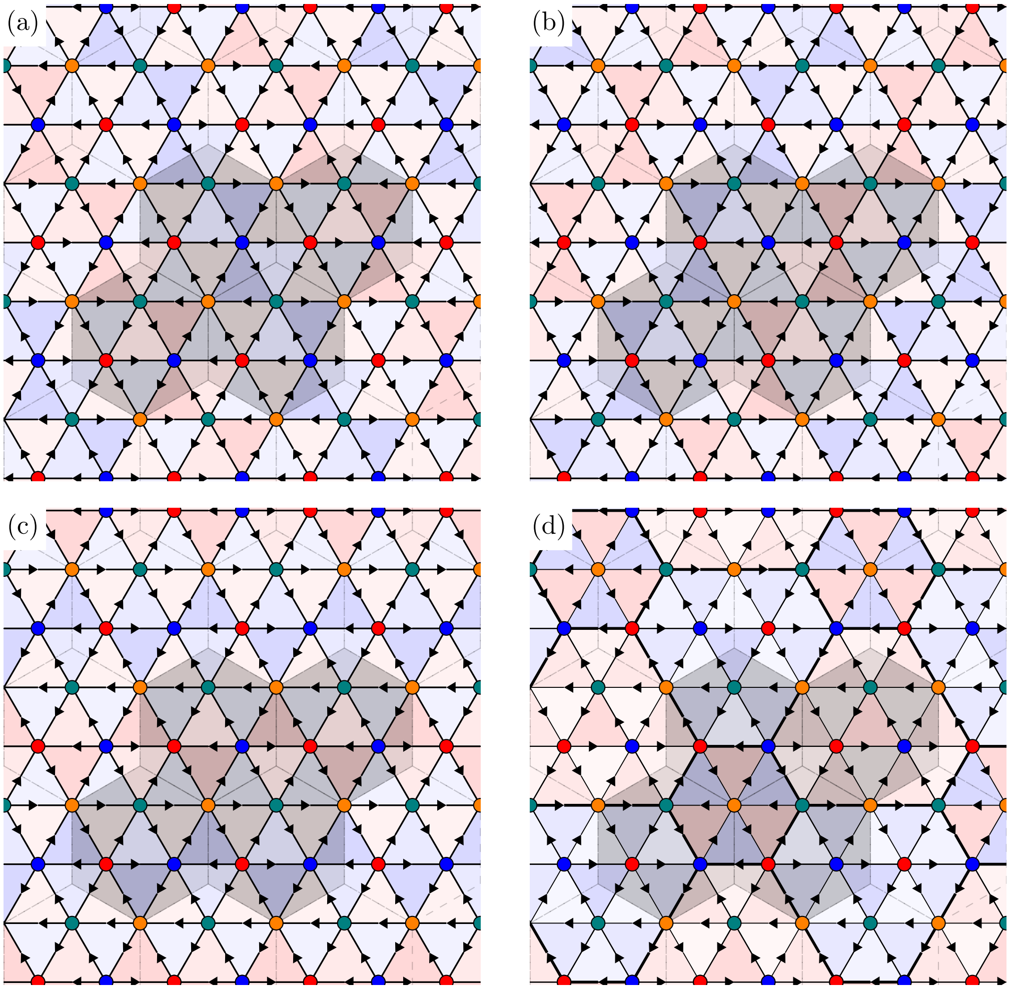

FIG. S7. The current patterns for Fig. 3(e) of the main text. They are in the irrep mM_3^- and have both currents flowing between V-V and V-Sb. The patterns (a),(b),(c) have ordering wave vectors $\boldsymbol{M}_1$, $\boldsymbol{M}_2$, and $\boldsymbol{M}_3$, respectively. The fourth pattern (d) is the superposition of the three patterns.

S3. PHENOMENOLOGICAL VS. MICROSCOPIC SUSCEPTIBILITIES

In this section, we microscopically calculated loop current propagators for various loop current patterns using the random phase approximation (RPA) and compare them with the phenomenological form written down in Eq. (3) of the main text. Both give very similar results for the pairing states, as determined by the linearized gap equation.

A. Microscopic computation of loop-current susceptibility

For the computation of a loop current susceptibility, we consider a loop current pattern defined by a fermion bilinear

$$\hat{J}^{\ell}_{\boldsymbol{q}} = \sum_{\boldsymbol{k}ab\sigma} \hat{c}^{\dagger}_{\boldsymbol{k},a,\sigma} J^{\ell}_{ab}(\boldsymbol{k}, \boldsymbol{k}+\boldsymbol{q}) \hat{c}_{\boldsymbol{k}+\boldsymbol{q},b,\sigma} \tag{S7}$$

Here, $\ell \in \{1, 2, 3\}$ labels the three different current patterns within the M-point irrep. The definition of the loop current susceptibility is (n labels the unit cell):

$$\chi^{\ell\ell'}_{\mathrm{LC}}(\boldsymbol{R}_n, \tau; \boldsymbol{R}_{n'}) = -\langle T_\tau[\hat{J}^{\ell}_{n}(\tau)\hat{J}_{n'}]\rangle, \tag{S8}$$

which, in Fourier space, reads

$$\chi^{\ell\ell'}_{\mathrm{LC}}(\boldsymbol{q}, iq_0) = -\frac{1}{N}\int_0^\beta e^{iq_0\tau}\langle T_\tau[\hat{J}^{\ell}_{\boldsymbol{q}}(\tau)\hat{J}_{-\boldsymbol{q}}]\rangle \mathrm{d}\tau \tag{S9}$$

$$= -\frac{1}{\beta N}\langle J^{\ell}_{q} J^{\ell'}_{-q}\rangle \tag{S10}$$

whereby the second line is written in the path integral language, such that the imaginary time ordering is automatic. If computed with respect to the noninteracting Hamiltonian, this gives the bare susceptibility. To go beyond the bare susceptibility, we need to include corrections generated by the interactions between loop current modes. The minimal approach to do this is through RPA. For this purpose, we add to our Hamiltonian the following bare local loop current interaction:

$$\hat{H}_{\mathrm{int}} = -\frac{1}{2NV}\sum_{\boldsymbol{q},\ell} : \hat{J}^{\ell}_{\boldsymbol{q}} \hat{J}^{\ell}_{-\boldsymbol{q}} : \tag{S11}$$

One way of arriving at this interaction is by decomposing extended Hubbard interactions into various channels and then retaining only the relevant loop current channel that, by assumption, becomes dominant in our system. Since our current operators $\hat{J}^{\ell}_{\boldsymbol{q}}$ have units of energy, proportional to the hopping elements, we put the coupling constant V in the denominator. Hence, the effective interaction in this channel is $H_{\mathrm{int}} \propto \mathrm{hopping}^2/V$. Moving to the path integral formalism, we can write an imaginary time action for the theory:

$$S = \underbrace{\int_0^\beta \sum_{\boldsymbol{k}ab\sigma} \bar{c}_{\boldsymbol{k}\tau a\sigma}(\delta_{ab}\partial_\tau + H_{ab}(\boldsymbol{k}))c_{\boldsymbol{k}\tau b\sigma}\mathrm{d}\tau}_{S_0} + \underbrace{\frac{-1}{2NV}\int_0^\beta \sum_{\boldsymbol{q},\ell} J^{\ell}_{\boldsymbol{q}} J^{\ell}_{-\boldsymbol{q}}\mathrm{d}\tau}_{S_{\mathrm{int}}} \tag{S12}$$

$$= \underbrace{\sum_{kab\sigma} \bar{c}_{ka\sigma}(-ik_0\delta_{ab} + H_{ab}(\boldsymbol{k}))c_{kb\sigma}}_{S_0} + \underbrace{\frac{-1}{2\beta NV}\sum_{q,\ell} J^{\ell}_{q} J^{\ell}_{-q}}_{S_{\mathrm{int}}} \tag{S13}$$

We now recall the Hubbard-Stratonovich transformation:

$$\int \exp\left(-\frac{1}{\beta NV}\sum_q \left[\frac{1}{2}|\Phi_q|^2 - \Phi_q J_{-q}\right]\right)\mathcal{D}\Phi = \exp\left(\frac{1}{2\beta NV}\sum_q J_q J_{-q}\right). \tag{S14}$$

Here, $\Phi_q = \Phi^*_{-q}$ is the real bosonic field of the loop current mode. This allows us to rewrite the grand partition

function as follows:

$$\mathcal{Z} = \int e^{-S_0 - S_{\text{int}}} \mathcal{D}[\bar{c}, c] \tag{S15}$$

$$= \int e^{-S_0} \exp\left(\frac{1}{2\beta NV} \sum_{q\ell} J_q^\ell J_{-q}^\ell \right) \mathcal{D}[\bar{c}, c] \tag{S16}$$

$$= \int e^{-S_0} \exp\left(-\frac{1}{\beta NV} \sum_{q\ell} \left[\frac{1}{2} |\Phi_q^\ell|^2 - \Phi_q^\ell J_{-q}^\ell \right] \right) \mathcal{D}\Phi \mathcal{D}[\bar{c}, c] \tag{S17}$$

The loop current susceptibility in the J-language is a 4-fermion correlation function that is directly related to a 2-point correlation in the Φ-language. To show this, we write the correlation function as a functional derivative:

$$\chi_{\text{LC}}^{\ell\ell'}(q) = -\frac{1}{\beta N} \langle J_q^\ell J_{-q}^{\ell'} \rangle \tag{S18}$$

$$= -\frac{1}{\beta N \mathcal{Z}} \int e^{-S_0} \exp\left(-\frac{1}{2VN\beta} \sum_{q\ell} |\Phi_q^\ell|^2 \right) \exp\left(\frac{1}{\beta NV} \sum_{q\ell} \Phi_q^\ell J_{-q}^\ell \right) J_q^\ell J_{-q}^{\ell'} \mathcal{D}\Phi \mathcal{D}[\bar{c}, c] \tag{S19}$$

$$= -\frac{1}{\mathcal{Z}} \int e^{-S_0} \exp\left(-\frac{1}{2VN\beta} \sum_{q\ell} |\Phi_q^\ell|^2 \right) \left[\beta NV^2 \frac{\delta}{\delta \Phi_{-q}^\ell} \frac{\delta}{\delta \Phi_q^{\ell'}} \exp\left(\frac{1}{\beta NV} \sum_{q\ell} \Phi_q^\ell J_{-q}^\ell \right) \right] \mathcal{D}\Phi \mathcal{D}[\bar{c}, c] \tag{S20}$$

Through two integrations by parts, this can be rewritten as

$$\chi_{\text{LC}}^{\ell\ell'}(q) = -\frac{1}{\mathcal{Z}} \int e^{-S_0} \left(-V\delta_{\ell\ell'} + \frac{1}{\beta N} \Phi_{-q}^{\ell'} \Phi_q^\ell \right) \exp\left(-\frac{1}{2VN\beta} \sum_{q\ell} |\Phi_q^\ell|^2 \right) \exp\left(\frac{1}{\beta NV} \sum_{q\ell} \Phi_q^\ell J_{-q}^\ell \right) \mathcal{D}\Phi \mathcal{D}[\bar{c}, c] \tag{S21}$$

$$= V\delta_{\ell\ell'} - \frac{1}{\beta N} \langle \Phi_q^\ell \Phi_{-q}^{\ell'} \rangle \tag{S22}$$

The RPA susceptibility is then obtained very easily by computing the $\langle \Phi_q^\ell \Phi_{-q}^{\ell'} \rangle$ correlation function at leading order in the coupling. To do this, we integrate out the electrons and then truncate the effective action for Φ at quadratic order. Doing this, we find that

$$\mathcal{Z} = \int e^{-S_{\text{eff}}[\Phi]} \mathcal{D}\Phi \tag{S23}$$

$$e^{-S_{\text{eff}}[\Phi]} = \int e^{-S} \mathcal{D}[\bar{c}, c] \tag{S24}$$

$$= \exp\left(-\frac{1}{2VN\beta} \sum_{q\ell} |\Phi_q^\ell|^2 \right) \int \exp\left(\sum_{kab} \bar{c}_{ka\sigma} [\mathcal{G}_0^{-1}(\boldsymbol{k}, ik_0)]_{ab} c_{kb\sigma} + \frac{1}{\beta NV} \sum_{qk\ell ab\sigma} \Phi_{k-q}^\ell \bar{c}_{ka\sigma} J_{ab}^\ell(\boldsymbol{k}, \boldsymbol{q}) c_{qb\sigma} \right) \mathcal{D}[\bar{c}, c] \tag{S25}$$

$$= \exp\left(-\frac{1}{2VN\beta} \sum_{q\ell} |\Phi_q^\ell|^2 \right) \int \exp\left(\sum_{kqab\sigma} \bar{c}_{ka\sigma} \left(\delta_{kq} [\mathcal{G}_0^{-1}(\boldsymbol{k}, ik_0)]_{ab} + \frac{1}{\beta NV} \sum_{\ell} \Phi_{k-q}^\ell J_{ab}^\ell(\boldsymbol{k}, \boldsymbol{q}) \right) c_{qb\sigma} \right) \mathcal{D}[\bar{c}, c] \tag{S26}$$

$$= \exp\left(-\frac{1}{2VN\beta} \sum_{q\ell} |\Phi_q^\ell|^2 \right) \det\left(\delta_{kq} [\mathcal{G}_0^{-1}(\boldsymbol{k}, ik_0)]_{ab} + \frac{1}{\beta NV} \sum_{\ell} \Phi_{k-q}^\ell J_{ab}^\ell(\boldsymbol{k}, \boldsymbol{q}) \right) \tag{S27}$$

whereby we define the (inverse of the) noninteracting Green function to be

$$[\mathcal{G}_0^{-1}(\boldsymbol{k}, ik_0)]_{ab} = ik_0 \delta_{ab} - H_{ab}(\boldsymbol{k}). \tag{S28}$$

This allows us to expand the effective action in terms of powers of Φ and get that

$$S_{\text{eff}}[\Phi] = \frac{1}{2V\beta N}\sum_{q\ell}|\Phi_q^\ell|^2 - \log\det\left(\delta_{kq}[\mathcal{G}_0^{-1}(\boldsymbol{k}, ik_0)]_{ab} + \frac{1}{\beta NV}\sum_\ell \Phi_{k-q}^\ell J_{ab}^\ell(\boldsymbol{k},\boldsymbol{q})\right) \tag{S29}$$

$$= \frac{1}{2V\beta N}\sum_{q\ell}|\Phi_q^\ell|^2 - \operatorname{tr}\log\left(\delta_{kq}[\mathcal{G}_0^{-1}(\boldsymbol{k}, ik_0)]_{ab} + \frac{1}{\beta NV}\sum_\ell \Phi_{k-q}^\ell J_{ab}^\ell(\boldsymbol{k},\boldsymbol{q})\right) \tag{S30}$$

$$= \frac{1}{2V\beta N}\sum_{q\ell}|\Phi_q^\ell|^2 - \operatorname{tr}\log\left(\delta_{kq}\delta_{ac} + \frac{1}{\beta NV}\sum_\ell \Phi_{k-q}^\ell [\mathcal{G}_0(\boldsymbol{k}, ik_0)]_{ab} J_{bc}^\ell(\boldsymbol{k},\boldsymbol{q})\right) + \text{const}, \tag{S31}$$

whereby the constant arises after we factor out the noninteracting Green function. Using the standard Taylor expansion

$$\log(\mathbb{1} + X) = X - \frac{X^2}{2} + \frac{X^3}{3} + \cdots, \tag{S32}$$

we truncate the effective action at second order, and find that

$$S_{\text{eff}}^{(2)}[\Phi] = \frac{1}{2V\beta N}\sum_{q\ell}|\Phi_q^\ell|^2 + \frac{1}{2(\beta NV)^2}\sum_{\substack{kq\ell\ell' \\ aba'b'}} \Phi_{-q}^\ell \Phi_q^{\ell'} [\mathcal{G}_0(\boldsymbol{k}, ik_0)]_{ab} J_{ba'}^\ell(\boldsymbol{k}, \boldsymbol{k}+\boldsymbol{q}) [\mathcal{G}_0(\boldsymbol{k}+\boldsymbol{q}, ik_0 + iq_0)]_{a'b'} J_{b'a}^{\ell'}(\boldsymbol{k}+\boldsymbol{q}, \boldsymbol{k}) \tag{S33}$$

$$= \frac{1}{2\beta NV}\sum_{q\ell\ell'} \Phi_{-q}^\ell [\mathcal{D}_{\text{RPA}}^{-1}(\boldsymbol{q}, iq_0)]_{\ell\ell'} \Phi_q^{\ell'}. \tag{S34}$$

This defines the (inverse of the) loop current boson's RPA-dressed propagator:

$$[\mathcal{D}_{\text{RPA}}^{-1}(\boldsymbol{q}, iq_0)]_{\ell\ell'} = \underbrace{\delta_{\ell\ell'}}_{[\mathcal{D}_0^{-1}(\boldsymbol{q}, iq_0)]_{\ell\ell'}} + \frac{1}{\beta NV}\sum_k \operatorname{tr}[\mathcal{G}_0(\boldsymbol{k}, ik_0) J^\ell(\boldsymbol{k}, \boldsymbol{k}+\boldsymbol{q}) \mathcal{G}_0(\boldsymbol{k}+\boldsymbol{q}, ik_0 + iq_0) J^{\ell'}(\boldsymbol{k}+\boldsymbol{q}, \boldsymbol{k})]. \tag{S35}$$

In terms of the eigenvectors $|u_n(\boldsymbol{k})\rangle$ and eigenvalues $\xi_n(\boldsymbol{k})$ of the Hamiltonian $H(\boldsymbol{k})$, the bare fermionic Green function is given by $\mathcal{G}_0(\boldsymbol{k}, ik_0) = \sum_n (ik_0 - \xi_n(\boldsymbol{k}))^{-1} |u_n(\boldsymbol{k})\rangle \langle u_n(\boldsymbol{k})|$. After inserting this and evaluating the fermionic Matsubara sum, we find that

$$[\mathcal{D}_{\text{RPA}}^{-1}(\boldsymbol{q}, iq_0)]_{\ell\ell'} = \delta_{\ell\ell'} - \frac{1}{NV}\sum_{\boldsymbol{k}mn} M_{\ell\ell'}^{mn}(\boldsymbol{k}, \boldsymbol{k}+\boldsymbol{q}) \frac{-(f_{\boldsymbol{k},m} - f_{\boldsymbol{k}+\boldsymbol{q},n})}{\xi_m(\boldsymbol{k}) - \xi_n(\boldsymbol{k}+\boldsymbol{q}) + iq_0}, \tag{S36}$$

where $M_{\ell\ell'}^{mn}(\boldsymbol{k}, \boldsymbol{k}+\boldsymbol{q}) = \langle u_m(\boldsymbol{k})| J^\ell(\boldsymbol{k}, \boldsymbol{k}+\boldsymbol{q}) |u_n(\boldsymbol{k}+\boldsymbol{q})\rangle \langle u_n(\boldsymbol{k}+\boldsymbol{q})| J^{\ell'}(\boldsymbol{k}+\boldsymbol{q}, \boldsymbol{k}) |u_m(\boldsymbol{k})\rangle$ and $f_{\boldsymbol{k},m} = [e^{\beta\xi_m(\boldsymbol{k})} + 1]^{-1}$ is the Fermi-Dirac distribution. For $\ell = \ell'$, $M_{\ell\ell}^{mn}(\boldsymbol{k}, \boldsymbol{k}+\boldsymbol{q}) \geq 0$ and the second term on the right-hand side is therefore always negative, pushing the dressed Green function towards divergence as we approach condensation of the corresponding loop current. This RPA propagator for loop currents can now be inserted into the formula for the linearized gap equation, replacing the phenomenological propagator $g^2\mathcal{D}_{\text{LC}}(\boldsymbol{q}) \to \mathcal{D}_{\text{RPA}}(\boldsymbol{q})$ from all equations in the main text and also the "Methods" section.

Lastly, let us note that the 2-point Φ correlation function at the RPA level equals:

$$\langle \Phi_q^\ell \Phi_{-q}^{\ell'} \rangle = \beta NV [\mathcal{D}_{\text{RPA}}(\boldsymbol{q}, iq_0)]_{\ell\ell'}. \tag{S37}$$

Plugging this into the formula (S22) for the LC susceptibility, we obtain

$$\chi_{\text{LC,RPA}}^{\ell\ell'}(q) = V\delta_{\ell\ell'} - \frac{1}{\beta N}\beta NV[\mathcal{D}_{\text{RPA}}(\boldsymbol{q}, iq_0)]_{\ell\ell'} \tag{S38}$$

$$= V\delta_{\ell\ell'} - V[\mathcal{D}_{\text{RPA}}(\boldsymbol{q}, iq_0)]_{\ell\ell'}. \tag{S39}$$

B. Plots of the phenomenological propagator vs. RPA propagator

To compare the phenomenological LC propagator with the RPA propagator, we plot both in the entire Brillouin zone. For convenience, we repeat the phenomenological propagator from the main text, Eq. (3), which was given by

$$[\mathcal{D}_{\mathrm{LC}}(\boldsymbol{q})]_{\ell\ell'} = \frac{\delta_{\ell\ell'}}{r + (1-r) f(\boldsymbol{q} - \boldsymbol{M}_\ell)}, \tag{S40}$$

$$f(\boldsymbol{q}) = \frac{2}{3} - \frac{2}{9}\big(\cos(\boldsymbol{q}\cdot\boldsymbol{a}_1) + \cos(\boldsymbol{q}\cdot\boldsymbol{a}_2) + \cos(\boldsymbol{q}\cdot\boldsymbol{a}_3)\big). \tag{S41}$$

This propagator we directly compare with the RPA propagator from the previous section.

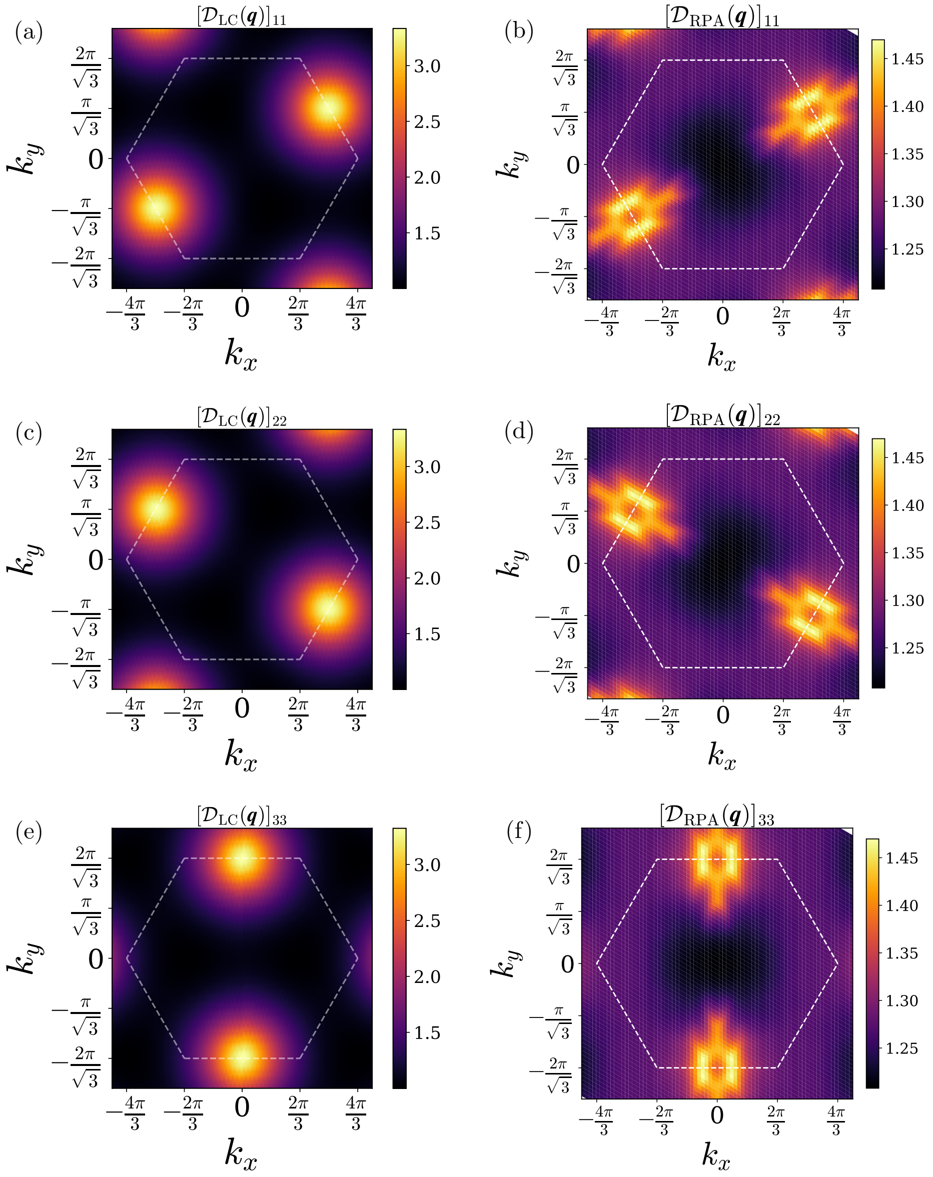

FIG. S8. Comparison of the phenomenological LC propagators (first column) with the RPA LC propagators (second column). In panels (a),(b), it is the susceptibility peaking at $\boldsymbol{M}_1$. Similarly for panels (c),(d) the peak is at $\boldsymbol{M}_2$ and for panels (e),(f) the peak is at $\boldsymbol{M}_3$. The peaks in the RPA propagators are not precisely at the M-points, but rather are closeby. This weak incommensurability is due to the imperfect nesting of the tight-binding model band structure. In spite of this difference, the pairing instabilities do not change.

S4. PROJECTED COOPER INTERACTION

In this section, we show the singlet and triplet interactions generated by all current interactions. Two of these are repeated from the main text but we include them here for completeness. In Fig. S9 we have all singlet interactions, and in Fig. S10 we have all triplet interactions. Fig. S9(a)–(c) are the same as main text Fig. 4(a)–(c), whereas Fig. S9(g)–(i) are the same as main text Fig. 4(d)–(f). Looking at this Fig. S9 for the 5 types of LC patterns, we make the following observations:

- If we compare the even vs. odd parity V-V loop current patterns, e.g. Fig. S9(a) vs. (j), we first see that there is still nearly no interaction to the Γ-pocket, and hence no $s^{\pm}$ pairing.
- Again comparing even vs. odd parity V-V loop current patterns Fig. S9(a) vs. (j), we see in (a) there is a strong interaction between M-points. However, this specific interaction is suppressed in (j) because of the odd parity of the current pattern. In spite of this, there is strong interaction to wave vectors close to the M-point, although not right at the M-point. This makes the leading pairing symmetry, $d + id$, less obvious than in the even parity case. Nonetheless, our calculations show that $d + id$ prevails in both the even and odd parity V-V LC patterns.
- We can also compare the two different patterns which are V-Sb, one of which is parity even, and the other parity odd. In particular, if we examine Fig. S9(g) vs. (m), we see that there is a large interaction between the outer Fermi surface and the inner Fermi surface. This fact is true regardless of the parity, and tells us that the favoured pairing symmetry is likely to be $s^{\pm}$, which is indeed what is found through the linearirzed gap equation calculation.

We therefore conclude that the pairing symmetry depends fundamentally on the microscopic loop current pathway, as this strongly determines the Fermi surface regions that feel the largest interaction.

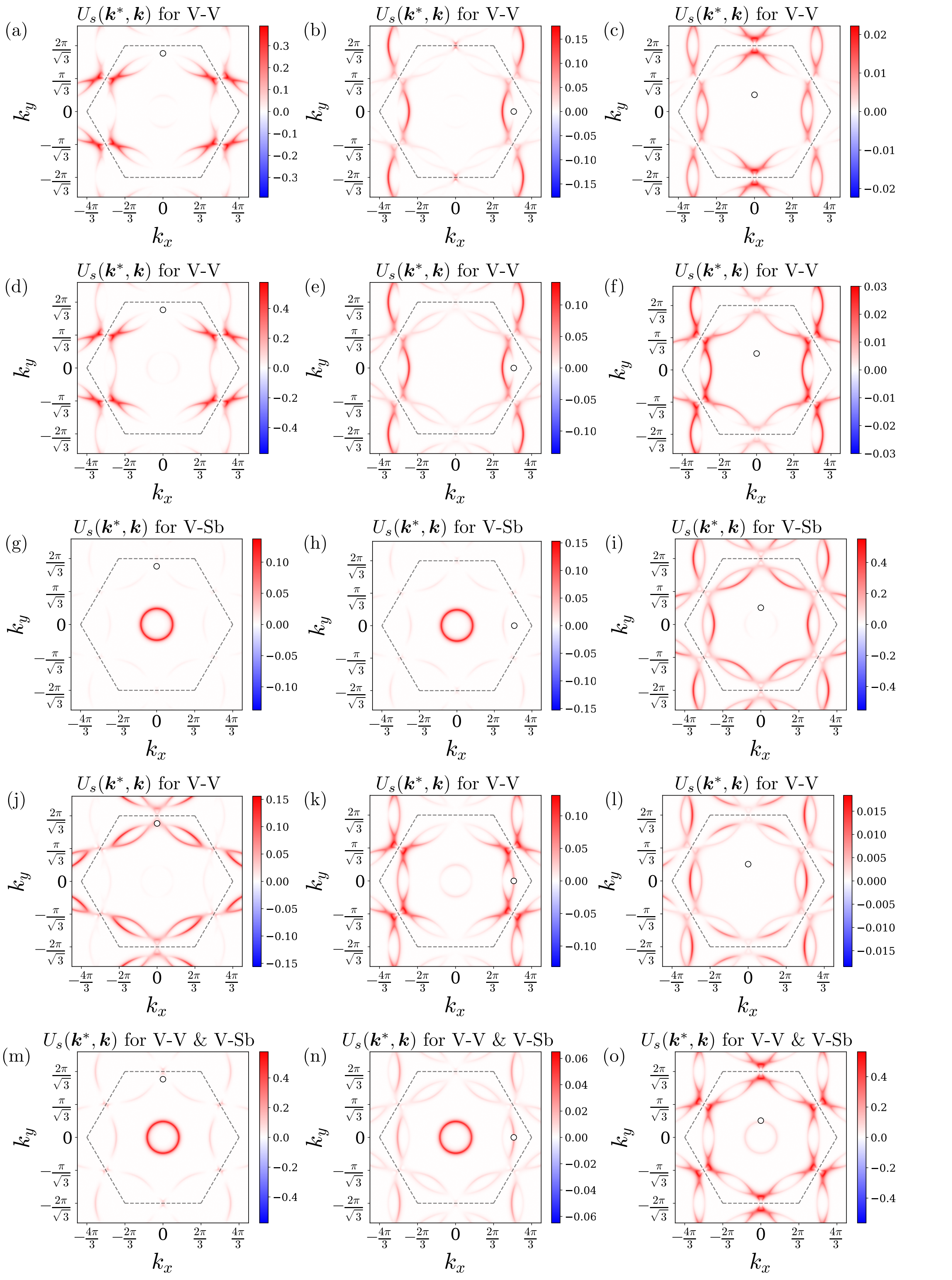

FIG. S9. Singet interactions. (a)-(c) are for main text Fig. 3(a). (d)-(f) are for main text Fig. 3(b). (g)-(i) are for main text Fig. 3(c). (j)-(l) are for main text Fig. 3(d). (m)-(o) are for main text Fig. 3(e). The key feature is that the first, second, and fourth rows (which are from V-V LC patterns) have very small interaction with the Γ pocket, whereas lines 3 and 5 (which have a V-Sb component) have strong interaction with the Γ pocket. This is the reason why the pathway is the determining factor for the resulting pairing symmetry.

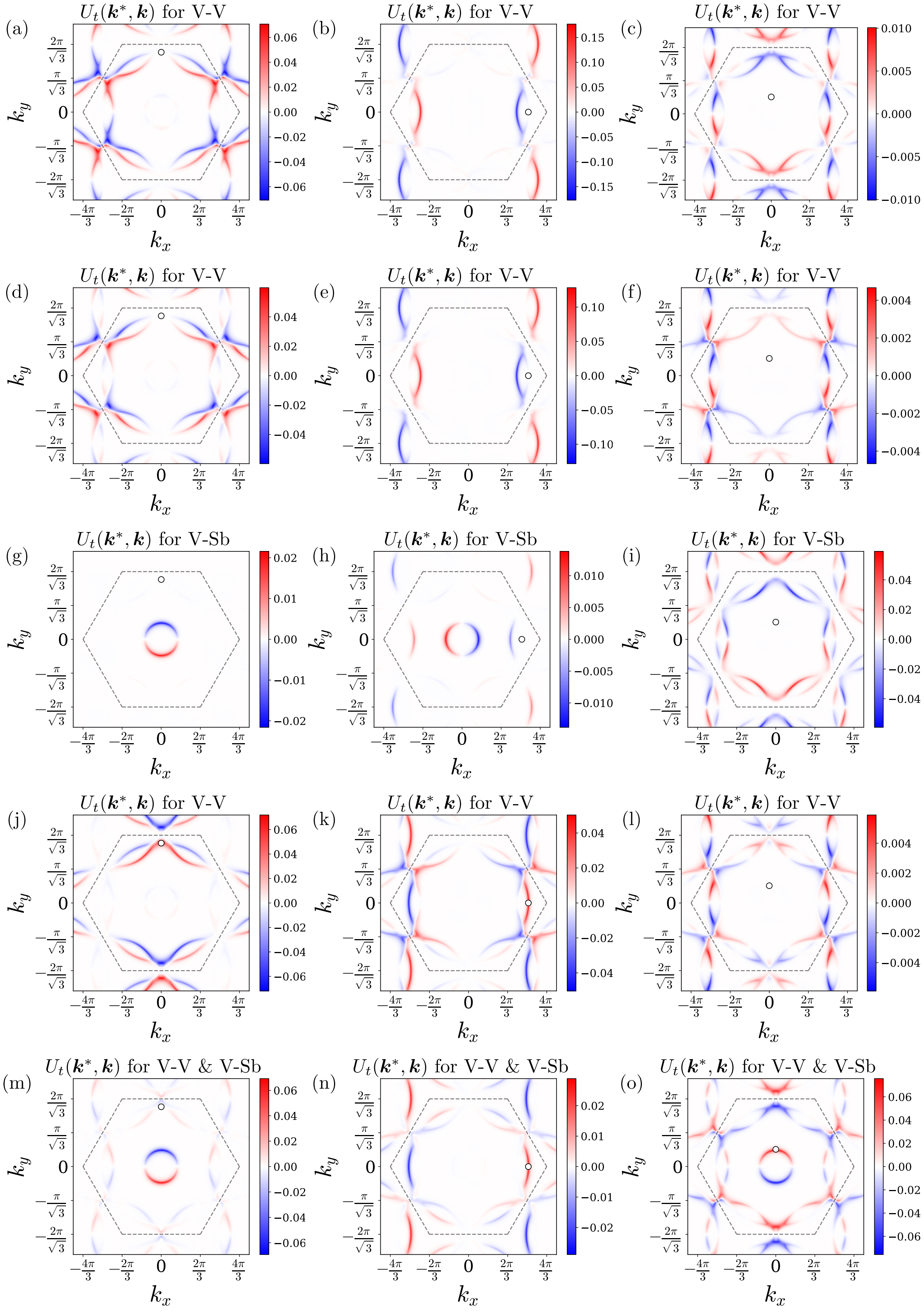

FIG. S10. Triplet interactions. (a)-(c) are for main text Fig. 3(a). (d)-(f) are for main text Fig. 3(b). (g)-(i) are for main text Fig. 3(c). (j)-(l) are for main text Fig. 3(d). (m)-(o) are for main text Fig. 3(e).

[1] X. Feng, K. Jiang, Z. Wang, and J. Hu, Science Bulletin **66**, 1384 (2021).
[2] H.-J. Yang, H. S. Kim, M. Y. Jeong, Y. B. Kim, M. J. Han, and S. Lee, SciPost Physics Core **6**, 008 (2023).
[3] J.-W. Dong, Z. Wang, and S. Zhou, Physical Review B **107**, 045127 (2023).
[4] X. Feng, Y. Zhang, K. Jiang, and J. Hu, Physical Review B **104**, 165136 (2021).
[5] H. Li, Y. B. Kim, and H.-Y. Kee, Physical Review Letters **132**, 146501 (2024).
